# Prevalence and Factors Associated with Intimate Partner Physical Violence Among Pregnant Women in Rwanda

**DOI:** 10.24248/eahrj.v9i2.853

**Published:** 2025-12-24

**Authors:** Annet Murungi, Hinda Ruton, Noel Gahamanyi, Joseph Ntaganira

**Affiliations:** a Field Epidemiology Training Program, Department of Epidemiology and Biostatistics, School of Public Health, College of Medicine and Health Sciences, University of Rwanda, Kigali-Rwanda; b Department of Epidemiology and Biostatistics, School of Public Health, College of Medicine and Health Sciences, University of Rwanda, Kigali-Rwanda; c Biology Department, School of Science, College of Science and Technology, University of Rwanda, Kigali Rwanda; d Microbiology Unit, National Reference Laboratory, Rwanda Biomedical Centre, Kigali, Rwanda

## Abstract

**Background:**

Intimate partner physical violence (IPPV) during pregnancy is a public health concern and a breach of human rights. Physical violence has major consequences for the mother, fetus, and newborn child. This study aimed at investigating the prevalence and factors associated with IPPV among pregnant women in Rwanda.

**Methods:**

This cross-sectional study used secondary data from the Rwanda Demographic Health Survey 2019–2020. A total of 1,849 women aged 15–49 years were included in this study. Descriptive statistics were used to determine the prevalence and frequency of different attributes while a multivariate logistic regression model was used to identify the factors associated with IPPV among pregnant women. Adjusted Odds Ratio (AOR) and 95% Confidence Interval (CI) were used to report the magnitude of the association between different variables and IPPV among pregnant women. Stata 16 was used for analysis and survey commands were applied to all analyses.

**Results:**

The prevalence of IPPV among pregnant women in Rwanda was 4.5% and the associated factors were the partner's alcohol drinking habits, drinks and sometimes gets drunk (AOR 3.68, 95% CI (1.3–10.1), drinks and gets drunk often (AOR 14.67, 95% CI (5.2–41.4), number of living children 3–5 (AOR 2.64 95% CI (1.2–5.6), and polygamous couple (AOR 2.81 95% CI (1.2–6.4).

**Conclusion:**

IPPV against pregnant women is still present in Rwanda. Having a male partner that drinks alcohol and gets drunk, polygamy, and having 3–5 children were factors associated with this IPPV. It is important to address the issue of substance abuse, refine social norms, and attitudes that promote gender inequalities through several stereotypes in families, empower women, and encourage them to use contraceptives. These findings emphasize the need for evidence-based policies and integration of prevention measures in the maternal health services to prevent IPPV against mothers and protect their unborn children.

## BACKGROUND

Violence against women is a breach of human rights, a public health concern, and a hindrance to sustainable development.^1^ Intimate partner violence (IPV) comprises of physical, sexual, and emotional abuse and obsessive or being controlled by an intimate partner.^2^ Intimate partner physical violence (IPPV) is defined as acts that can cause physical harm to the victim including but not limited to slapping, kicking, hitting with a fist or anything else, beating or dragging, chocking, burning one intentionally, threatening with or actually having a gun, knife or other weapon used, by an intimate partner.^1^ According to national surveys in Rwanda, physical violence comprises actions of beating, hitting, kicking, choking, burning, or threats using a weapon.^3^ Some indicators of IPV include physical injuries such as lacerations, contusions, and fractures often reported by patients as home accidents.^4^ Currently, the World Health Organization (WHO) and UN Women estimate that one in three women suffer a minimum of one type of intimate partner violence (IPV) once in their life.^5^

The worldwide lifetime prevalence of violence (physical and/or sexual) against married or everpartnered women of 15 to 49 years of age was 27% in 2018.^6^ In Africa, 36.6% of women experienced lifetime physical and/or sexual IPV among everpartnered women and the Sub-Saharan-Africa (SSA) countries have been reported as top sites of violence against women.^7^ In SSA, Ethiopia and Ghana reported the prevalence of 20.6% and 5%, respectively.^8^ In Rwanda, previous studies have reported IPV with prevalence varying from 33.6–35.1%.^9,10^ The report from the National Institute of Statistics of Rwanda (NISR) reported that 41.4% of women and 17.9% of men believe that wife beating is justified for different reasons.^10^

A number of efforts and programs have been put in place to reduce IPV in SSA. These include programs created for women empowerment, men and boys’ engagement, and changing community norms that turn a blind eye to violence and support male supremacy.^11^ The government of Rwanda (GoR) enacted a law for the prevention and punishment of Gender-Based Violence (GBV) in 2008 with six months minimum imprisonment sentence.^12^ Several interventions like establishing Isange one-stop centers receiving those experiencing violence, clubs against GBV in secondary schools and universities, community-level prevention committees, Parents’ evening meetings were used by the GoR to fight against GBV and IPV by promoting awareness, identify and help victims of GBV and IPV.^12,13^ Isange one-stop centers provide medical care, psychosocial support, short-term shelter and legal assistance to victims of IPV and child abuse.^12^

Violence against women during pregnancy may lead to serious medical and social problems.^14^ Studies have shown that pregnant women who experienced IPV were at a higher risk of premature rapture of membrane, vaginal bleeding, ^15^ preterm labor, unwanted pregnancy, antenatal hospitalization, ^16^ poor weight gain, ^17^ postpartum depression, ^18^ poor mental health quality of life, ^19^ pre-eclampsia, hypertension and spontaneous abortion.^20^ A study showed that women suffering from IPV have a doubled risk of delayed prenatal visit till the third trimester compared to those who did not.^21^ Also, IPV during pregnancy can lead to severe and deadly outcomes like deaths, low weight at birth (LBW), ^22^ perinatal mortality and neonatal mortality.^23^ Studying IPPV during pregnancy is crucial as pregnancy creates unique biological, psychological, and social conditions that affect two individuals (the mother and the fetus) at once. This makes the consequences of violence more severe and far-reaching than at other times in a woman's life. Therefore, understanding the prevalence of IPPV during pregnancy and the associated factors may have important reproductive and maternal health implications and inform important interventions in Rwanda.

There are several factors associated with IPV. These include lower education status of the partners, rural residency, choice of partner by women only, ^24^ age at first marriage, women without formal education, ^8^ being in a polygamous marriage or union, multiparity, witnessing maternal abuse in childhood, ^25^ increased number of children in the family, separated or divorced marital status, ^26^ lack of contraceptive use, ^21^ and employment status of the woman.^27^ Studies conducted in Rwanda showed that IPV among pregnant women were associated with occupation of the partner, women involvement in decision making about their health and purchases, ^13^ being a woman living with Human Immunodeficiency Virus (HIV), male partner who drinks alcohol or with other partners.^9^ In Rwanda, there is a shortage of literature on IPPV among pregnant women because no recent studies capturing current trends, especially given social changes, legal reforms, or increased awareness around gender-based violence. Also, most of the studies are cross-sectional or retrospective and thus offering limited information on long-term effects of the IPPV. Therefore, this study aimed at assessing the prevalence of IPPV and associated factors among pregnant women in Rwanda.

## METHODS

### Study Design

A cross-sectional study utilizing secondary data on 1,849 women from 13,000 households aged between 15–49 years who answered and filled the domestic violence module during the Rwanda Demographic and Health Survey (RDHS 2019–2020). The RDHS is part of the global DHS project which promotes global understanding of health and population tendencies in several countries through providing technical assistance for the implementation of surveys. The DHS collects a wide range of objective and self-reported data with a strong focus on indicators of fertility, reproductive health, maternal and child health, mortality, nutrition, and self-reported health behaviors among adults. The study was carried out through multistage cluster sampling of villages and households, with stratification of all the 30 districts. Data collection was done from 09/11/2019 to 20/07/2020 but it was halted by the nationwide lockdown during the COVID-19 pandemic between 21/03/2020 to 07/06/2020. The interviewers were given specific training to carry out safe and confidential interviews. Interviews were conducted face-to-face in the respondent's homes and women were interviewed about physical violence experiences during pregnancy.

### Study setting

This study was conducted in Rwanda classified by the World Bank as a low-income country. The country contains five regions (Kigali city and four provinces) which is home to around 12 million people.^28^ The average household consists of 4.4 persons and the gross domestic product per capita was US dollars 816.^29^

### Sample Size

The study sample was based on women of age 15 to 49 years with 1,849 respondents considered for the RDHS 2019–2020 survey.

### Inclusion Criteria

All women aged 15 to 49 years who were pregnant in the last five years before the study, with complete data on the outcome variable.

### Data Collection Method

Secondary data of women aged 15–49 years were downloaded using Stata 16 from the measure DHS website and access was granted through a written letter sent via email.

### Quantitative Variables

Age of wife, age of partner, woman's education, partner's education, woman's employment status, partner's employment status, age at first marriage, number of living children, attitude towards husband beating wife, household wealth quintile, residence; (urban/rural), marital status, decision making involvement at home for women about her health, woman involved in decision making about her earnings, partner alcohol consumption, woman's father beat mother, polygamous couple, lack of contraceptive use.

### Outcome Variable

Intimate partner physical violence during pregnancy (Yes/No).

### Data Analysis

Collected data were analyzed using Stata 16. Descriptive statistics (percentages, and frequencies) were computed to analyze sociodemographic, reproductive characteristics, history of IPPV and related characteristics of the study participants. To test the significance of association between sociodemographic characteristics, reproductive characteristics, IPPV history and related characteristics, with IPPV during pregnancy, bivariate logistic regression at 95% confidence interval was applied with *p*<.05 regarded as statistically significant. For multivariate logistic regression analysis, factors with *p*<.05 in the design-based chi-square for categorical variables were considered. Collinearity was assessed for all variables and manual backward stepwise regression was used to build the final model. To record the magnitude of association between sociodemographic characteristics, reproductive characteristics, IPPV history and related characteristics with IPPV among pregnant women, Adjusted Odds Ratio (AOR) and 95% confidence interval were used. For all analyses, sampling weights and adjustments for clustering and stratification of observations were applied. To adjust for oversampling of urban primary sampling units and alteration for clustering of observations within primary sampling units (PSUs) and districts’ stratification, svyset commands to apply inverse probability weights were used.

### Ethical Consideration

The Institutional Review Board (IRB) of the University of Rwanda approved this study and granted the ethical permit to conduct the research (CMHS/1RB/115/2023). As secondary data were utilized, informed consent was not necessary. However, a confidentiality agreement was signed. To preserve confidentiality and anonymity, deidentified data were downloaded with consent.

## RESULTS

### Sociodemographic Characteristics of Study Participants

A total of 1,849 women were included in this study. The majority of women were aged 30–39 years (789; 42.7%), 1,545 (83.6%) of women were from rural areas, 1,392 (75.3%) were in union/living with a man, 1,303 (70.5%) had informal employment, 942 (68.2%) had primary level education, 1,186 (64.1%) had a partner with primary level education, and 1,028 (55.6%) had a partner with informal employment (Table 1).

**TABLE 1:** Sociodemographic Characteristics of the Women who Experienced Intimate Partner Physical Violence (IPPV) During Pregnancy

Sociodemographic characteristics of the participants	Did not experience IPPV Violence in pregnancy % (95% CI)	Experienced IPPV in pregnancy % (95% CI)
Respondent's age group (n=1,849)		
15–19 (n=43)	95.8[75.4–99.4]	4.2[0.6–24.6]
20–24 (n=204)	94.5[86.9–97.8]	5.5[2.2–13.1]
25–29 (n=301)	96.9[94.5–98.2]	3.1[1.8–5.5]
30–39 (n=789)	95.6[93.8–96.9]	4.4[3.1–6.2]
40–49 (n=512)	95.0[91.8–97.0]	5.0[3.0–8.2]
Residence (n=1,849)		
Urban (n=304)	95.4[94.0–96.4]	4.6[3.6–6.0]
Rural (n=1,545)	95.5[94.2–96.5]	4.5[3.5–5.8]
Marital status (n=1,849)		
Never in union (n=189)	97.8[86.3–99.7]	2.2[0.3–13.7]
Currently in union/living with a man (n=1392)	95.3[93.9–96.3]	4.7[3.7–6.1]
Formerly in union/living with a man (n=268)	95.5[94.2–96.5]	4.5[3.5–5.8]
Partner's Occupation (n=1,849)		
Not employed (n=556)	93.3[90.2–95.4]	6.7[4.6–9.8]
Formal (n=265)	97.0[90.7–99.1]	3.0[0.9–9.3]
Informal (n=1,028)	96.4[94.9–97.4]	3.6[2.6–5.1]
Respondent Occupation (n=1,849)		
Not employed (n=267)	95.3[89.7–97.9]	4.7[2.1–10.3]
Formal (n=279)	96.7[93.3–98.4]	3.3[1.6–6.7]
Informal (n=1,303)	95.3[93.8–96.5]	4.7[3.5–6.2]
Woman's educational level (n=1382)		
No Education (n=185)	95.7[91.7–97.8]	4.3[2.2–8.3]
Primary (n=942)	95.7[94.1–96.8]	4.3[3.2–5.9]
Secondary and Above (n=255)	96.9[90.2–99.0]	3.1[1.0–9.8]
Husband/partner's education level (n=1849)		
No Education (n=247)	95.0[91.3–97.1]	5.0[2.9–8.7]
Primary (n=1186)	95.4[94.0–96.5]	4.6[3.5–6.0]
Secondary and Above (n=416)	94.4[89.5–97.1]	5.6[2.9–10.5]
Wealth index combined (n=1,849)		
Poorest (n=384)	95.7[93.3–97.2]	4.3[2.8–6.7]
Poorer (n=364)	96.0[93.7–97.6]	4.0[2.4–6.3]
Middle (n=359)	94.0[90.4–96.3]	6.0[3.7–9.6]
Richer (n=392)	95.9[92.6–97.7]	4.1[2.3–7.4]
Richest (n=350)	96.0[90.6–98.4]	4.0[1.6–9.4]
Partner's Age group (n=1392)		
Below 30 (n=219)	98.8[96.3–99.6]	1.2[0.4–3.7]
30–39 (n=592)	95.6[93.6–97.0]	4.4[3.0–6.4]
40 and above (n=580)	95.2[92.4–97.0]	4.8[3.0–7.6]

### The Magnitude of IPPV against Pregnant Women

The overall prevalence of IPPV among pregnant women in Rwanda was 4.5%. (83/1849) and women aged 20–24 had the highest prevalence of 5.5%.

Based on all characteristics of the study sample, the highest prevalence was observed among women with a partner who drink alcohol and get drunk often (16.1%), and those in polygamous partnership (10.5%) (Table 2).

**TABLE 2:** IPPV Related Characteristics

IPPV related characteristics of the participants	Did not experience IPPV Violence in pregnancy % (95% CI)	Experienced IPPV in pregnancy % (95% CI)
Polygamous Couple (n=1,392)		
No (n=1,268)	97.2[96.0–98.0]	2.8[2.0–4.0]
Yes (n=124)	89.5[78.8–95.1]	10.5[4.9–21.2]
Husband's drinking habits (n=1,660)		
Doesn't drink (n=605)	97.9[95.7–99.0]	2.1[1.0–4.3]
Drinks, never drunk (n=223)	99.3[94.9–99.9]	0.7[0.1–5.1]
Drinks, sometimes drunk (n=623)	95.1[93.0–96.6]	4.9[3.4–7.0]
Drinks, often drunk (n=209)	83.9[77.2–88.9]	16.1[11.1–22.8]
Respondent decision about Earnings (n=807)		
No (n=70)	95.2[87.0–98.3]	4.8[1.7–13.0]
Yes (n=737)	97.1[95.6–98.1]	2.9[1.9–4.4]
Respondent decision about Health (n=1,392)		
No (n= 249)	96.3[92.9–98.1]	3.7[1.9–7.1]
Yes (n=1,143)	96.5[95.0–97.6]	3.5[2.4–5.0]
Age at first Marriage (n=1,660)		
20 years and below (n=700)	94.6[92.5–96.1]	5.4[3.9–7.5]
21 years and above (n=959)	95.8[93.9–97.1]	4.2[2.9–6.1]
Beating is justified (n=1,849)		
No (n=916)	95.2[92.9–96.7]	4.8[3.3–7.1]
Yes (n=933)	95.9[94.3–97.0]	4.1[3.0–5.7]
Father beat respondent's mother (n=1,726)		
No (n=1,064)	96.6[95.0–97.6]	3.4[2.4–5.0]
Yes (n=662)	93.9[91.2–95.8]	6.1[4.2–8.8]

### Reproductive characteristics of women who experienced IPPV during pregnancy

Women who don't use contraceptives had a higher prevalence of IPPV of 5.4% compared to women who use contraceptives 3.8%. Women who had (3–5) children had a higher IPPV prevalence 6.3% compared to women who had less than three children at 3.7%.

### Bivariate analysis of factors associated with IPPV among pregnant women

In the unadjusted model with bivariate analysis, the factors (sociodemographic, reproductive, history) which were statistically significant (*P* <.05) include: partner's age group of ≥ 40 years (*P*=.026), marital status: formerly in union (*P*<.001), partner's occupation: unemployed (*P*=.004), partner's alcohol drinking habits: sometimes drinks and gets drunk (*P*=.039), often drinks and gets drunk (*P*<.001), living children: 3–5 children (*P*=.036), and polygamous couple (*P*=.002) (Table 4).

Women with a partner aged ≥ 40 (COR 3.96, 95% CI (1.18–13.30) were 3.96 times more likely to suffer from IPPV during pregnancy compared to those with partners aged ≤30 years. Also, women who were formerly in union/living with a man (COR 4.23, 95% CI (2.70–6.69) were 4.23 times more likely to suffer IPPV during pregnancy, compared to those who were currently in union/living with a man.

Having an unemployed partner (COR 4, 95% CI (1.52–8.70) had four times higher odds of IPPV during pregnancy compared to having a partner with formal employment.

Women with a partner who drinks alcohol and sometimes gets drunk (COR 2.38, 95% CI (1.05–5.41) and women with a partner who drink alcohol and often gets drunk (COR 8.87, 95% CI (3.82–20.60) were 2.38 and 8.87 times, respectively, more likely to suffer IPPV during pregnancy compared to those with a partner who doesn't drink alcohol.

Having 3 to 5 living children (COR 1.67, 95% CI (1.04–2.69) had 1.67 times more risk of IPPV during pregnancy than having less than 3 children. Women in a polygamous partnership (COR 4.01, 95% CI (1.63–9.87) were 4.01 times more likely to suffer IPPV during pregnancy compared to those who were not (Table 3).

**TABLE 3:** Bivariate Analysis of Sociodemographic, Reproductive, IPPV Related Characteristics Among Pregnant Women

Characteristics	COR	95%CI	*P value*
Sociodemographic			
Age group (n=1,849)			
15–19 (n=43)	Reference		
20–24 (n=204)	1.16	[0.14–9.57]	.891
25–29 (n=301)	1.21	[0.15–9.51]	.856
30–39 (n=789)	1.35	[0.18–10.20]	.769
40–49 (n=512)	1.64	[0.21–12.52]	.633
Partner's Age group (n=1392)			
Below 30 (n=219)	Reference		
30–39 (n=592)	3.1	[0.93–10.4]	.067
40 and above (n=580)	3.96	[1.18–13.30]	.026
husband/partner's education level (n=1849)			
No Education (n=247)	2.32	[0.76–7.01]	.138
Primary (n=1186)	1.81	[0.70–4.62]	.223
Secondary and Above (n=416)	Reference		
marital status (n=1,849)			
Never in union (n=189)	0	[0.02–1.29]	.089
Currently in union/living with a man (n=1392)	Reference		
Formerly in union/living with a man (separated/divorced/widowed) (n=268)	4.23	[2.70–6.69]	<.001
Respondent Occupation (n=1,849)			
Not employed (n=267)	1	[0.43–2.30]	.999
Formal (n=279)	Reference		
Informal (n=1,303)	1.08	[0.56–2.09]	.808
Woman's educational Level (n=1382)			
No Education (n=185)	1	[0.69–3.22]	.312
Primary (n=942)	1	[0.72–2.37]	.378
Secondary and Above (n=255)	Reference		
Partner's Occupation (n=1,849)			
Not employed (n=556)	4	[1.52–8.70]	.004
Formal (n=265)	Reference		
Informal (n=1,028)	1.67	[0.70–3.96]	.25
wealth index combined (n=1,849)			
Poorest (n=384)	Reference		
Poorer (n=364)	1	[0.46–1.79]	.789
Middle (n=359)	1.4	[0.70–2.79]	.337
Richer (n=392)	1	[0.45–2.06]	.911
Richest (n=350)	1	[0.33–2.56]	.874
Residence			
Rural (n=1,545)	1.34	[0.74–2.45]	.338
Urban (n=304)	Reference		
IPPV related characteristics			
Husband's drinking habits (n=1,660)			
Doesn't drink (n=605)	Reference		
Drinks, never drunk (n=223)	0.35	[0.04–2.81]	.32
Drinks, sometimes drunk (n=623)	2.38	[1.05–5.41]	.039
Drinks, often drunk (n=209)	8.87	[3.82–20.60]	
<.001			
Age at first Marriage (n=1,660)			
20 years and below (n=700)	Reference		
21 years and above (n=959)	0.67	[0.43–1.03]	.069
Respondent decision about Earnings (n=807)			
No (n=70)	1.56	[0.53–4.60]	.418
Yes (n=737)	Reference		
Respondent decision about Health (n=1,392)			
No (n= 249)	1.09	[0.54–2.19]	.819
Yes (n=1,143)	Reference		
Beating is justified (n=1,849)			
No (n=916)	Reference		
Yes (n=933)	0.96	[0.62–1.48]	.842
Father beat respondent's mother (n=1,726)			
No (n=1,064)	Reference		
Yes (n=662)	1.52	[0.97–2.37]	.068
Polygamous Couple (n=1,392)			
No (n=1,268)	Reference		
Yes (n=124)	4.01	[1.63–9.87]	.002
Reproductive characteristics			
living children (n=1,849)			
Less than 3 (n=830)	Reference		
3–5 (n=634)	1.67	[1.04–2.69]	.036
Above 5 (n=384)	1.22	[0.64–2.32]	.538
Use of contraceptive (n=1,849)			
No (n=750)	0.69	[0.41–1.19]	.184
Yes (n=1,099)	Reference		

**TABLE 4:** Multivariate Logistic Regression Analysis of Factors Associated with IPPV Among Pregnant Women in Rwanda

Factors	AOR	95% CI	*P value*
Husband's drinking habits (n=1,660)			
Doesn't drink (n=605)	Reference		
Drinks, never drunk (n=223)	0.35	0.04–2.81	.32
Drinks, sometimes drunk (n=623)	3.68	1.3–10.1	.011
Drinks, often drunk (n=209)	14.67	5.2–41.4	<.001
Living children (n=1,849)			
Less than 3 (n=830)	Reference		
3–5 (n=634)	2.64	1.2–5.6	.011
Above 5 (n=384)	2.13	0.8–5.6	.126
Polygamous Couple (n=1,392)			
No (n=1,268)	Reference		
Yes (n=124)	2.81	1.2–6.4	.014

### Multivariate analysis of factors associated with IPPV among pregnant women

After adjusting for confounding variables, IPPV was associated with partner's alcohol drinking habits, number of living children and polygamy. From this analysis, women with partners that drink alcohol and sometimes get drunk and those who drink alcohol and often get drunk had 3.68 times (AOR 3.68, 95% CI (1.3–10.1) and 14.67 times (AOR 14.67, 95%% CI (5.2–41.4), respectively had higher odds of IPPV during pregnancy compared to those with partners who don't drink alcohol and those who drink but never get drunk. Furthermore, women who had 3–5 living children (AOR 2.64 95% CI (1.2–5.6) had 2.64 times higher odds of IPPV during pregnancy compared to those who had <3 children. Also, women in a polygamous partnership (AOR 2.81 95% CI (1.2–6.4) had 2.81 times higher odds of IPPV during pregnancy compared to women who were not (Table 4).

## DISCUSSION

The prevalence of IPPV towards pregnant women in Rwanda was 4.5%. This concurs with a prevalence of 5% reported in Ghana but lower than 10% and 11.9% and 20.6% reported in Kenya and Ethiopia, respectively.^25,8,30^ The lower rate IPPV in Rwanda may be due to the difference in the study design because Rwanda and Ghana used national cross-sectional surveys while in Ethiopia and Kenya, both studies were cross-sectional, but facility based using systematic random sampling technique. The lower rate could also be due to the multi-sectoral efforts by the Government of Rwanda to fight against violence against women, through various measures such as imprisoning the perpetrators, setting up one stop centers for the victims to report and seek all the necessary help.^12,13^ The IPPV towards pregnant women in Rwanda was associated with intimate partner alcohol consumption (consuming alcohol and get drunk). This is similar to other studies conducted in Kenya^25^ and Ethiopia.^8^ This could be attributable to reduced control of emotions after drinking alcohol and thus becoming aggressive towards their partners.^7^ Alcohol consumption can limit one's ability to resolve conflicts peacefully and this may complicate minor misunderstandings that can lead to violence.

In addition, IPPV towards pregnant women in Rwanda was also associated with polygamy. This is consistent with a previous study conducted in Rwanda, ^9^ Kenya ^25^ and Pakistan.^31^ Polygamous relationships usually involve jealousy and hatred among the partners involved ^32^ which creates tension and this usually results into aggression and thus physical violence. Nowadays, polygamy is not accepted in various communities due to religious beliefs.^7^ Cultural beliefs and norms that accept polygamy may also reinforce the gender inequality and patriarchal practices which may make physical violence against females tolerable in society, thus making it difficult for the victims to report it due to fear of judgement.

The IPPV towards pregnant women was significantly associated with the number of living children (3–5 children). This is similar to a study conducted in Uganda.^33^ Having more than three children requires a lot of attention from the parents, especially the mothers. This reduces the time to care for the partner (husband) which may negatively affect the relationship between couples, thus increasing the likelihood of violence.

Unlike other studies, the level of education was not significantly associated with IPPV in this study. Other studies indicated that women with no formal education and those with primary level education were more likely to be assaulted by their partners during pregnancy.^8,15^ This is because less educated women lack knowledge and skills for women empowerment that are usually acquired through formal education. This could leave them vulnerable and susceptible to violence from their partners.

Although partner's level of education was not significant in our study in the multivariate analysis, the bivariate analysis showed that pregnant women whose intimate partners had no formal education were 2.78 times more likely to be abused than those whose partners had a high school education. other studies have shown that men who did not attain secondary school^34^ or partners who were uneducated^8^ were more likely to be perpetrators of violence towards their intimate partners.

The current study faced a few limitations. First, it used data obtained through self-reporting which could lead to misreporting due to fear of social stigma by women who participated in the survey. Second, the study was cross-sectional, and thus causality cannot be established. Third, recall bias could negatively affect the findings as women may not accurately remember events of violence earlier in pregnancy and emotional trauma may distort or suppress memories, affecting reporting. Despite such limitations, the study findings can inform decision makers on right interventions to reduce IPPV and improve the health of affected women.

## CONCLUSION

This study reported on prevalence of IPPV among pregnant women in Rwanda. IPPV towards pregnant women in Rwanda remains a public health issue. The study highlighted that IPPV was associated with having a male partner who drinks and gets drunk, having three to five children, and being a polygamous couple. We recommend the Government of Rwanda to adopt strategies to curb alcohol consumption, increase awareness campaigns on the benefits of family planning and monogamy. IPPV screening should be integrated into antenatal care services to provide early care and better reporting. Community based interventions should be implemented to curb alcohol misuse and polygamy in society.
